# Premotor cortex is hypoactive during sustained vowel production in individuals with Parkinson’s disease and hypophonia

**DOI:** 10.3389/fnhum.2023.1250114

**Published:** 2023-10-23

**Authors:** Jordan L. Manes, Ajay S. Kurani, Ellen Herschel, Angela C. Roberts, Kris Tjaden, Todd Parrish, Daniel M. Corcos

**Affiliations:** ^1^Department of Speech, Language, and Hearing Sciences, Boston University, Boston, MA, United States; ^2^Ken and Ruth Davee Department of Neurology, Northwestern University, Chicago, IL, United States; ^3^Department of Radiology, Northwestern University, Chicago, IL, United States; ^4^Brain and Creativity Institute, University of Southern California, Los Angeles, CA, United States; ^5^School of Communication Sciences and Disorders, Western University, London, ON, Canada; ^6^Canadian Centre for Activity and Aging, Western University, London, ON, Canada; ^7^Department of Computer Science, Western University, London, ON, Canada; ^8^Department of Communication Sciences and Disorders, Northwestern University, Evanston, IL, United States; ^9^Department of Communicative Disorders and Sciences, University at Buffalo, Buffalo, NY, United States; ^10^Department of Physical Therapy and Human Movement Sciences, Northwestern University, Chicago, IL, United States

**Keywords:** Parkinson’s disease, hypophonia, voice, premotor cortex, speech, fMRI, phonation

## Abstract

**Introduction:**

Hypophonia is a common feature of Parkinson’s disease (PD); however, the contribution of motor cortical activity to reduced phonatory scaling in PD is still not clear.

**Methods:**

In this study, we employed a sustained vowel production task during functional magnetic resonance imaging to compare brain activity between individuals with PD and hypophonia and an older healthy control (OHC) group.

**Results:**

When comparing vowel production versus rest, the PD group showed fewer regions with significant BOLD activity compared to OHCs. Within the motor cortices, both OHC and PD groups showed bilateral activation of the laryngeal/phonatory area (LPA) of the primary motor cortex as well as activation of the supplementary motor area. The OHC group also recruited additional activity in the bilateral trunk motor area and right dorsal premotor cortex (PMd). A voxel-wise comparison of PD and HC groups showed that activity in right PMd was significantly lower in the PD group compared to OHC (p < 0.001, uncorrected). Right PMd activity was positively correlated with maximum phonation time in the PD group and negatively correlated with perceptual severity ratings of loudness and pitch.

**Discussion:**

Our findings suggest that hypoactivation of PMd may be associated with abnormal phonatory control in PD.

## 1. Introduction

The majority of individuals with Parkinson’s disease (PD) experience adverse changes to their speech and voice at some point throughout the disease course (Sapir, 2014). The constellation of motor speech symptoms in PD, referred to collectively as “hypokinetic dysarthria,” include monopitch, monoloudness, reduced stress, imprecise consonants, inappropriate silences, short rushes of speech, harsh voice, breathy voice, low pitch, and variable rate. Among the most prevalent changes to the parkinsonian voice is the development of hypophonia – a condition characterized by reduced loudness or “soft speech” (Duffy, 2019). The physiology of hypophonia includes deficits in both laryngeal function and respiratory support for speech breathing (Solomon and Hixon, 1993; Huber et al., 2003; Hammer et al., 2013; Huber and Darling-White, 2017). However, at the cortical level, it is not clear how reduced vocal intensity relates to changes in the activity of the motor cortices (i.e., primary motor cortex, premotor cortex, and SMA).

The reduction of vocal intensity in hypophonia appears to mirror the hypokinesia (reduced movement amplitude) observed in PD limb movements, and may reflect scaling deficits seen in PD (Sapir, 2014). Within the framework of the classic rate model of PD, hypokinetic and bradykinetic movements are purported to arise from reduced thalamocortical excitation of the motor cortices following the degeneration of dopaminergic cells in the substantia nigra pars compacta and subsequent dysregulation of the cortico-basal ganglia pathways. Thus, if the reduced vocal amplitude observed in hypophonia is indeed hypokinetic in nature, it should follow that motor cortical activity is hypoactive during phonation. However, reduced voice intensity in PD does not appear to have a consistent or robust response to dopaminergic therapy (Daniels et al., 1996; Kompoliti et al., 2000; Skodda et al., 2010; Fabbri et al., 2017). Moreover, existing neuroimaging studies of speech production in PD have reported mixed findings with respect to motor cortical activation, making it difficult to discern whether reduced vocal intensity is explicitly linked to reduced activity in the motor cortices (Pinto et al., 2004; Rektorova et al., 2007; Pinto et al., 2011; Arnold et al., 2014; Baumann et al., 2018; Narayana et al., 2020). The variability across studies may be in part due to differences in the clinical presentation of the participants (e.g., whether participants presented with voice symptoms at the time of the study). In addition, some of these findings may be specific to the speech paradigms used (e.g., overt vs. covert speech paradigms; connected vs. single word production).

Employing a task that is specifically phonatory in nature, such as sustained phonation of a vowel, may help to establish whether hypophonia is related to hypoactivation in the motor cortices. The existing imaging literature on PD speech has focused more broadly on speech production using overt sentence reading (Rektorova et al., 2007; Arnold et al., 2014), covert sentence reading (Baumann et al., 2018), and word production (Pinto et al., 2011) tasks. However, speech production tasks in healthy adults have been shown to recruit additional regions of the cortex that are not found during sustained phonation alone (Ozdemir et al., 2006). While sentence production tasks may provide a more global picture of hypokinetic dysarthria in PD and any associated changes in functional communication, they may not be sufficient for differentiating the physiological mechanisms of voice dysfunction from changes in articulation. Using a phonatory task, such as sustained vowel production, can help to disentangle mechanisms of hypophonia from other PD speech characteristics and provide clearer insight into which neural systems are driving changes in voice output.

In healthy adults, fMRI studies of vowel production/phonation have shown activity in primary motor cortex (Ozdemir et al., 2006; Soros et al., 2006; Brown et al., 2008; Grabski et al., 2013; Correia et al., 2020; Eichert et al., 2020; Brown et al., 2021), premotor cortex (Brown et al., 2008; Grabski et al., 2013), and supplementary motor area (SMA) (Soros et al., 2006; Brown et al., 2008; Grabski et al., 2013). Within the primary motor cortex, fMRI activity during phonation is most commonly reported in the bilateral laryngeal/phonatory area (LPA), also referred to as the ‘larynx motor cortex’. This functional area is located towards the middle/inferior region of the precentral gyrus and often includes two peaks of activation – the dorsolateral LPA and the ventromedial LPA (Brown et al., 2008; Bouchard et al., 2013; Belyk and Brown, 2017; Eichert et al., 2020; Belyk et al., 2021). In addition to the LPA, some fMRI studies of phonation have also reported activity in the trunk motor area, located on the superior aspect of the precentral gyrus (Guenther, 2016; Correia et al., 2020). For example, Correia et al. (2020) found additional bilateral activation in the trunk motor area when comparing voiced versus voiceless utterances. The authors proposed that activation of the trunk motor area is linked to the use of trunk muscles for respiratory control during phonation. Within the premotor cortex, it has been proposed that dorsal aspect (PMd), may be more involved with the coordination of pitch-related vocalizations and prosody, while the ventral aspect (PMv) may be more involved with the coordination of the phonetic or syllabic components of speech (Hickok et al., 2023). This model further suggests that the region of PMd responsible for coordinating vocal pitch provides input to the dorsal LPA (Hickok et al., 2023). Finally, SMA is involved with the initiation of voicing during phonation, as well as the initiation of speech production more broadly (Ziegler et al., 1997; Guenther, 2016; Jonas, 2019).

The purpose of this study was to (1) characterize the BOLD responses of older healthy controls (OHC) and PD patients with hypophonia during a sustained vowel task, (2) determine whether there are group differences in motor cortical activity between PD patients with hypophonia and OHC participants during sustained vowel production, and (3) determine the strength of the association between motor cortical activity and acoustic and perceptual measures of phonation. To accomplish this, we recruited a group of PD participants with hypophonia and a group of age-matched OHC participants to perform a sustained vowel production task while undergoing fMRI. In order to obtain high fidelity measures of voice intensity, participants also performed the same vowel production task outside of the scanner in a simulated MRI environment (Manes et al., 2021). We hypothesized individuals with PD and hypophonia would have significantly lower activity in the LPA, SMA, and premotor cortex compared to age-matched healthy controls and that our acoustic and perceptual measures of phonation would correlate BOLD activity in areas of hypoactivation.

## 2. Materials and methods

### 2.1. Participants

We recruited 15 OHC participants and 15 participants with PD to undergo fMRI scanning during vowel production. All participants provided informed consent for the study procedures, in accordance with Northwestern University’s guidelines. Of the initial 15 OHC and 15 PD participants recruited, 1 OHC and 2 participants with PD were excluded from the final analysis due to excessive motion or extracranial susceptibility artifacts in the fMRI data (for quality assurance criteria, refer to Section 2.6). The remaining 14 OHC and 13 PD participants were included in the final fMRI analysis.

All participants were right-handed, native English speakers between 40 and 80 years old. All participants had normal cognition, scoring ≥26 on the Montreal Cognitive Assessment (MoCA; Nasreddine et al., 2005) or ≥ 18 on the MoCA-Blind (if screened over the phone). Hearing thresholds for each participant were < 35 dB HL when assessed using bilateral pure tone average at 0.5, 1, and 2 kHz. Healthy control participants reported no history of speech, language, hearing, or neurological disorders. Participants in the PD group reported no history of speech, language, hearing, or neurological disorders aside from PD. There were no statistically significant group differences in age, sex, cognition, or hearing threshold (Table 1).

**Table 1:** Demographic features for the OHC and PD groups.

	Older healthy controls	Parkinson’s disease	*ꭓ*^2^-value	*t*-value	Value of *p*
N	14	13	–	–	–
Sex (male/female)	10/4	9/4	0.031	–	0.861
Age (years)	61.0 (±9.2)	62.2 (±8.9)	–	−0.35	0.728
DRS-2	141.43 (± 1.83)	140.62 (±1.89)	–	1.14	0.267
Hearing threshold (dB)^†^	17.44 (±7.83)	21.41 (±5.61)	–	−1.19	0.246

† Hearing threshold calculated using a binaural pure tone average across 500 Hz, 1,000 Hz, and 2000 Hz frequencies.

DRS-2, Mattis Dementia Rating Scale 2.

All PD participants were initially judged to have hypophonia by their referring movement disorders neurologist or by an MDS-UPDRS certified researcher trained to identify reduced voice intensity. The presence of hypophonia was later confirmed through perceptual ratings by three expert raters (2 speech-language pathologists and 1 research assistant) not involved in data collection (Section 2.3.2). None of the PD participants had completed the Lee Silverman Voice Treatment (LSVT) program (Ramig et al., 2001b) within the past 2 years. PD disease severity ranged from mild to moderate (Hoehn and Yahr Stage I-III), and all PD participants were being treated with antiparkinsonian medication. PD characteristics, including MDS-UPDRS ratings (Goetz et al., 2008), levodopa equivalent daily dose (Tomlinson et al., 2010), Hoehn and Yahr stage (Hoehn and Yahr, 1967; Goetz et al., 2004), and PD subtype (Stebbins et al., 2013) are reported in Table 2. Motor testing for the MDS-UPDRS Part III was conducted after 12-h overnight withdrawal from antiparkinsonian medications. fMRI testing was also completed after 12-h medication withdrawal in order to maximize the disease-state.

**Table 2:** Parkinson’s disease characteristics for PD participants.

**MDS-UPDRS**	
MDS-UPDRS total score	57.54
MDS-UPDRS part I	8.92
MDS-UPDRS part II	12.69
MDS-UPDRS part III (motor exam)	33.92
MDS-UPDRS part III (speech item)	1.23
MDS-UPDRS part IV	2
**Hoehn and Yahr**	
Stage 0	0 (0.0%)
Stage 1	1 (7.7%)
Stage 2	11 (84.6%)
Stage 3–5	1 (7.7%)
**TD/PIGD classification**	
TD	4 (30.8%)
PIGD	7 (53.8%)
Indeterminate	2 (15.4%)
**Side most affected**	
Left	8 (61.5%)
Right	5 (38.5%)
Symmetric	0 (0.0%)
**LEDD (mg)**	
Mean	747.92
Min, Max	120, 1563

MDS-UPDRS, Movement Disorders Society Unified Parkinson’s Disease Rating Scale, TD, tremor dominant, PIGD, postural instability and gait disturbance, LEDD, levodopa equivalent daily dose.

### 2.2. Sustained vowel production task

During the task, participants were presented with either a “+” symbol (Rest) or “Ah” (Vowel Production) block. During the “Ah” blocks, subjects were instructed to produce an /a/ vowel for approximately 3–5 s at their normal conversational loudness and repeat for the duration of the block. This self-paced paradigm was designed so that participants would rely on internal, rather than external cueing mechanisms for the initiation of each utterance. In-scanner recordings were used to verify task compliance and to measure the duration and number of vowels produced by the participant.

### 2.3. Acquisition and analysis of voice measures

#### 2.3.1. Collection of voice samples

Outside of the MRI scanner, we collected samples of maximum phonation time as well as voice sound pressure level (SPL) samples. Voice samples were recorded using a head mounted, unidirectional microphone (Shure SM10A) positioned 3 cm from the lower lip. The microphone was channeled through a pre-amplifier (ART Project Series USB Dual Pre) and then relayed onto a laptop computer for recording in Audacity (sampled at 44.1 kHz). For measures of maximum phonation time, participants were instructed to sustain an /a/ vowel for as long as possible in their normal conversational loudness. The productions were recorded in Audacity and manually marked for voice onset/offset and the maximum phonation time was reported in seconds. For measures of voice SPL, we used recordings collected outside of the scanner in which participants performed the sustained vowel production task. To capture performance that would be ecologically similar to our fMRI task, participants completed the sustained vowel task in a mock MRI scanner with fMRI acoustic noise presented over headphones at 90 dB. Recordings for the sustained vowel task were also collected with the participants seated upright and without the presence of fMRI noise. We calculated the difference in SPL between the Mock Scanner + Noise and Upright recording conditions to correct for group differences in the effects of the scanning environment on voice SPL (Manes et al., 2021). The order of fMRI and speech testing was counterbalanced to control for the effects of practice and vocal fatigue.

Inside of the MRI scanner, we collected acoustic recordings to monitor task compliance and to record the timing and duration of self-paced vowel productions. An MRI compatible microphone was mounted to the head coil and positioned ~1 cm from the lower lip. The audio signal was digitized using a Measurement Computing USB-1608G A/D board and processed in MATLAB. Using the volume trigger from the scanner, a template of the scanner noise was made during the time when the MR signal was reaching equilibrium and no patient sounds were made. Each subsequent time period had the template noise signature subtracted followed by temporal and frequency filtering to remove any scanner noise from the auditory signal (Sen and Parrish, 2007). The final result was output as a .wav file with time = 0 synchronized with the start of the experiment.

#### 2.3.2. Perceptual ratings of PD voice severity

Twelve of our thirteen PD participants received perceptual ratings of voice quality as part of a previous behavioral study (Manes et al., 2021). Perceptual ratings were performed using the Consensus Auditory-Perceptual Evaluation of Voice (CAPE-V; Kempster et al., 2009), which uses a 100 mm analogue scale to rate voice deviance in 5 perceptual domains (roughness, breathiness, strain, pitch, and loudness). Three expert raters listened to recordings of the participants producing /a/ and rated the perceived deviance for each perceptual domain.

#### 2.3.3. Acoustic analysis of voice measures (outside the scanner)

Maximum phonation time was analyzed using a one-way ANCOVA, with group defined as a fixed factor and age and hearing threshold defined as covariates. To examine the effect of group on voice SPL in our fMRI-analogous environment (Mock Scanner + MRI Noise), we fit a mixed effects (multi-level) models using the lmer command in the R package lme4 (version 1.1-21; Bates et al., 2015). In this model, random effects characterized the degree of difference across individual intercepts and slopes, while the fixed effects estimates were drawn from the average of these individual intercepts and slopes. Estimated *p* values were obtained *via* Kenward-Roger’s degrees of freedom method using the lmerTest package (version 3.1-0; Kuznetsova et al., 2017). We modeled the effect of group (OHC vs. PD) on vowel intensity, with fixed effects estimates of group, block, and their interactions. We coded factors using effects (simple) coding (Clopper, 2013). Group was coded as −0.5 for OHC and + 0.5 for PD participants. We treated block as a numeric variable to account for possible vocal decay across blocks, with the first block coded as zero. To account for individual differences in vowel intensity and vocal decay across blocks, we also included random intercepts and random linear slopes for each subject within the model. Random effects were estimated with an unstructured covariance matrix and restricted maximum likelihood estimation, using the Nelder–Mead optimization function.

Our previous study (Manes et al., 2021) suggested that our PD participants increased voice SPL to a greater degree than controls when laying supine and listening to MRI noise. Thus, to account for inter-subject differences in Lombard responses during fMRI, we calculated the difference in voice SPL between the “Mock Scanner + MRI Noise” (described above) and the “Upright” conditions in which participants were recorded while seated upright outside of the mock scanner (Manes et al., 2021). This “Lombard response” measure was entered as a covariate in the fMRI analysis.

#### 2.3.4. Acoustic analysis of task performance (inside the scanner)

Acoustic recordings from the MRI-compatible microphone were used to assess the durations and number of vowels produced in each task block. The onset and offset of each vowel production was manually labelled in Audacity.^1^ To test for group differences in task performance, we compared the mean vowel duration between PD and OHC groups using a two-sample *t*-test. We also compared the total number of vowels produced between PD and OHC groups using a two-sample *t*-test.

### 2.4. fMRI data acquisition

Imaging data were collected on a Siemens 3 T PRISMA-FIT MRI scanner using a 64-channel head coil. T1-weighted anatomical scans were collected in the sagittal plane using an MPRAGE GRAPPA sequence at a voxel resolution of 0.8 mm^3^ (TR = 2000 ms, TE = 2.99 ms, TI = 1,010 ms, flip angle = 8°, FOV = 256 mm, inversion time = 1,010 ms, BW = 240 Hz/pixel). T2-weighted anatomical scans were collected in the sagittal plane using a T2 SPACE sequence at a voxel resolution of 0.8mm^3^ (TR = 2,500 ms, TE = 566 ms, flip angle = 120°, FOV = 256 mm, BW = 710 Hz/pixel). For the fMRI scans, we employed a continuous scanning protocol. BOLD T2*-weighted functional scans were collected in 56 interleaved slices using a multiband acceleration factor of 2 and voxel size of 2mm^3^ (TR = 2000 ms, TE = 25 ms, flip angle = 80°, FOV = 208 mm). The sustained vowel production task was presented in the magnet room on a NordicNeuroLab LCD monitor using E-Prime and viewed through an angled mirror mounted to the head coil.

### 2.5. fMRI experimental procedure

All testing was performed at Northwestern University’s Center for Translational Imaging. During the scanning session, participants lay supine in the MRI scanner while wearing foam earplugs for hearing protection. An angled mirror was affixed to the head coil so that participants could view the stimulus prompt and an MRI-compatible microphone was affixed to the head coil to record the onset and offset of voicing. Prior to beginning the task, the participants were told that they would see either an “Ah” or “+” prompt on the screen. We instructed participants that whenever the “Ah” prompt appeared on the screen, they should produce an /a/ vowel for approximately 3–5 s at their normal conversational loudness and repeat at their own pace until the “Ah” prompt disappeared. We instructed the participants that whenever the “+” prompt appeared on the screen, they should stay silent and look at the crosshair. In order to limit movement of the head and jaw when vocalizing, we also instructed participants to keep their mouths slightly open through the duration of the task. After the instructions were given, the participants performed the sustained vowel production task during fMRI scanning. Total task time was 10 min, including ten vowel production blocks (30s each) and ten rest blocks (30s each).

### 2.6. fMRI data preprocessing

All fMRI data was processed and analyzed using SPM12 and AFNI tools. To be included in the statistical analysis, we required at least 7 min (210 time points) of usable data for each fMRI scan, with a framewise displacement (FD) < 0.5 mm and root-mean square signal change (DVARS) < 5%. Functional images underwent initial despiking and realignment to the reference frame (first time point). During realignment, 6 motion parameters were extracted. Additional motion correction was applied using the ART Repair Toolbox version 5b in SPM12.^2^ Corrupted volumes were ‘repaired’ using linear interpolation if the framewise displacement (FD) was greater than 0.5 mm. Functional images were then smoothed to a full-width-half maximum of 6 mm. Subject-level analysis of BOLD fMRI data was conducted in subject-space using a general linear model (GLM) in SPM12 before being normalized to MNI space for group-level statistical analysis. To perform the first-level GLM, we extracted the timing and duration of each self-paced vowel production using noise-attenuated, in-scanner microphone recordings. We then analyzed the data in a block design, with the block onset starting at the initiation of the first vocalization and ending at the end of the last vocalization. The subject-level GLM included the block timing and 6 motion parameters, with global normalization scaling applied. The first-level statistical results (Ah vs. Rest) were then normalized to MNI space using a multi-stage procedure that also accounts for geometric distortions due to magnetic field inhomogeneities (Jezzard and Balaban, 1995; Chambers et al., 2015). First, we co-registered the T2-weighted scans to the T1-weighted scan and then rigidly co-registered both T1- and T2-weighted scans to the first volume of the participant’s functional scan. Next, using a similar approach to Chambers et al. (2015), we nonlinearly warped the fMRI reference volume to the co-registered T2-weighted scan to estimate the distortions due to the magnetic field inhomogeneities (*Warp_1_*). Using AFNI’s auto_warp.py command, we then normalized the co-registered T1-weighted scan to the MNI 2009c symmetric template (Fonov et al., 2009) using combined affine and nonlinear warp transformations (*Warp_2_*). Finally, we combined *Warp_1_* and *Warp_2_* to calculate a single nonlinear warp transformation from fMRI space to MNI space. The combined transformation was then applied to the fMRI statistical maps in order to perform group-level statistical analysis in MNI space.

### 2.7. fMRI analysis of task-related activations

Group-level statistical analysis was conducted using SPM12. The second-level general linear model included the covariates of age, hearing threshold, SPL (within group), and Lombard response. Within each group, we generated task activation maps (Ah vs. Rest). We then calculated our between-group contrasts (OHC vs. PD). For all contrasts, we report results using a voxel-wise height threshold of *p* < 0.001, uncorrected and cluster extent threshold of *p* < 0.05, uncorrected (58 mm^3^). We also report cluster-level FDR-corrected value of *p*s for all contrasts.

## 3. Results

### 3.1. Perceptual ratings of PD voice severity

Deviance ratings across the five CAPE-V perceptual domains are reported in Table 3. Higher scores indicate more deviant voice characteristics. Table 3 suggests mild to moderate severity of voice characteristics of the PD group.

**Table 3:** Perceptual ratings of PD voice severity on the CAPE-V.

CAPE-V perceptual domains	Mean (±SD)
Roughness	40.04 (±16.30)
Breathiness	26.13 (±17.66)
Strain	29.46 (±8.75)
Pitch	18.79 (±9.42)
Loudness	23.67 (±11.74)

CAPE-V, consensus auditory-perceptual evaluation of voice, SD, standard deviation.

### 3.2. Acoustic voice measures (outside the scanner)

**Figure 1:**
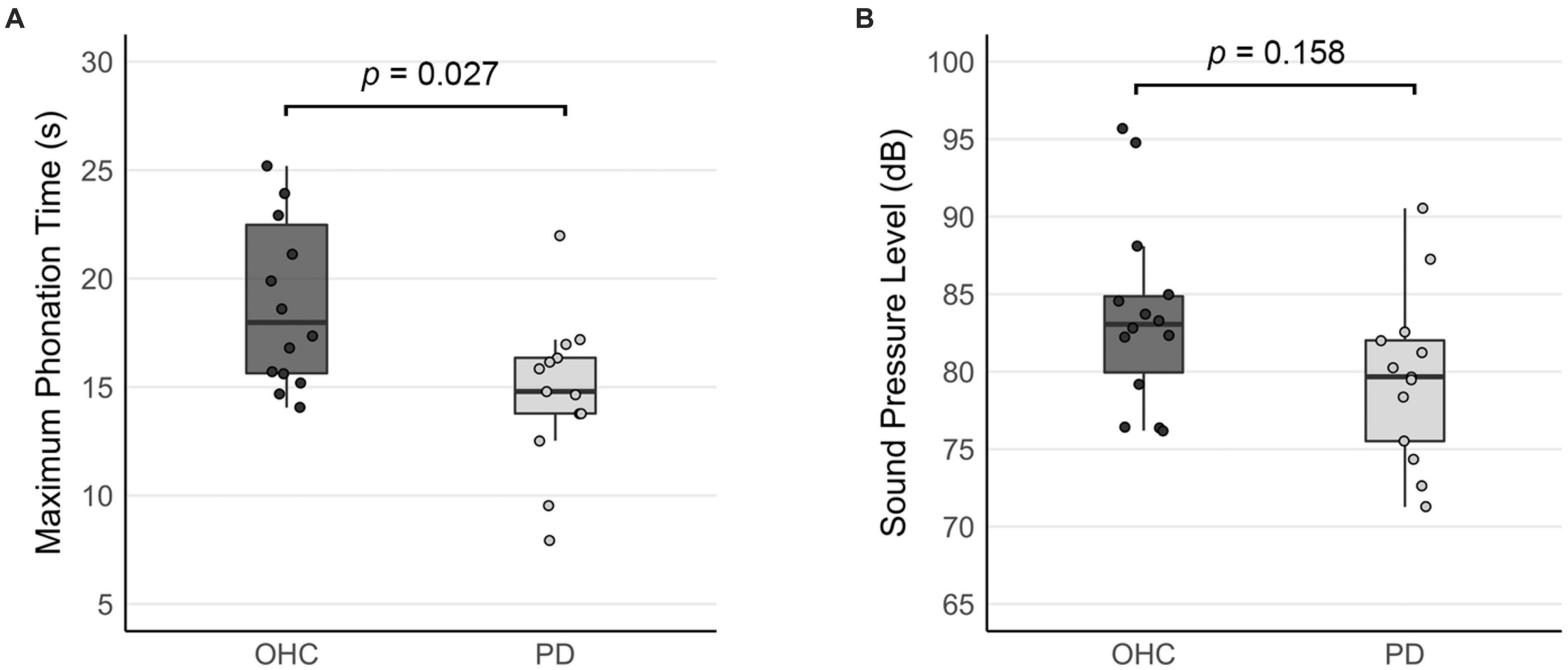
Comparison of acoustic measures between OHC and PD groups (outside the scanner). Bar graphs depict **(A)** mean maximum phonation times, and **(B)** the mean sound pressure levels collected when the sustained vowel production was performed in the mock MRI scanner. Error bars represent 95% confidence intervals. Whisker lines reflect 1.5 times the interquartile range. Dots represent data points for individual participants.

A one-way ANCOVA revealed a significant main effect of group for maximum phonation time (*F*(27) = 5.604, *p* = 0.027). Maximum phonation time was significantly shorter in the PD group compared to OHC (difference = 4.239, *p* = 0.027; Figure 1A).

Table 4 reports the linear mixed effects model for voice SPL in the mock MRI scanner. Although the mean SPL of the PD group was 4.712 dB lower than the OHC group (Figure 1B), our model output did not indicate a significant effect of PD on voice SPL (*β* = −3.29, SE = 2.26, *p* = 0.158). There was no significant effect of block, nor was there a significant group*block interaction.

**Table 4:** Output of linear mixed effects model for group effects on voice SPL.

	SPL					
Predictors	Estimates	Std. error	CI	Statistic	*p*	*df*
(Intercept)	81.51	1.13	79.19–83.84	72.17	<0.001	25.00
PD	−3.29	2.26	−7.94–1.36	−1.46	0.158	25.00
Block	−0.01	0.05	−0.11–0.09	−0.22	0.829	25.00
PD:Block	−0.09	0.10	−0.29–0.11	−0.91	0.369	25.00

Random effects	
σ^2^	1.01
τ_00 Sub_ID_	34.05
τ_11 Sub_ID.Block_	0.05
ρ_01 Sub_ID_	−0.07
ICC	0.97
N _Sub_ID_	27
Observations	270
Marginal *R*^2^/conditional *R*^2^	0.088/0.974

### 3.3. Task performance inside the scanner

**Figure 2:**
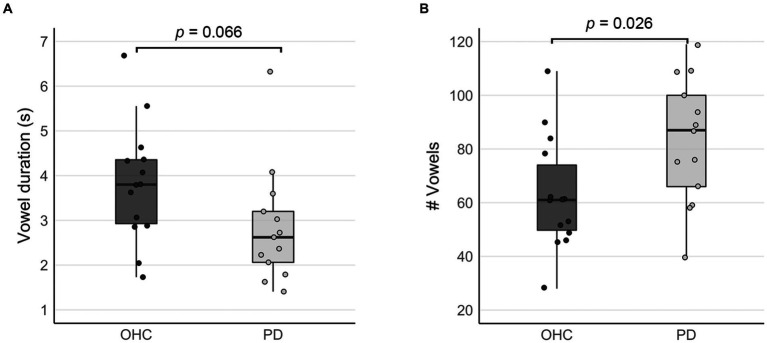
Comparison of self-paced task performance between OHC and PD groups (inside the scanner). Box plots depict **(A)** mean vowel durations, and **(B)** the mean number of vowels produced when performing the sustained vowel task in the MRI scanner. Whisker lines reflect 1.5 times the interquartile range. Dots represent data points for individual participants.

Due to the self-paced design of the sustained vowel task, we recorded the duration and number of vowels produced by each subject during the vowel production blocks. On average, participants in the OHC group sustained /a/ vowels for a mean duration of 3.82 s, while the mean duration for participants in the PD group was 2.85 s (Figure 2A). Despite the trend for shorter vowels in the PD group, there was no statistically significant group difference in vowel duration (*t* = 1.920, *p* = 0.066). Participants in the PD group produced significantly more vowels than the OHC group (OHC mean: 62.8 vowels, PD mean: 83.2 vowels, *t* = −3.71, *p* = 0.026; Figure 2B). Thus, overall, the vowels produced by the PD group tended to be of higher frequency and shorter duration. The total duration of vowel productions across the entire fMRI task was not statistically different between OHC and PD groups (OHC mean: 215.8 s, PD mean: 212.1 s, *t* = 0.335, *p* = 0.741).

### 3.4. Bold activity during sustained vowel production

**Figure 3:**
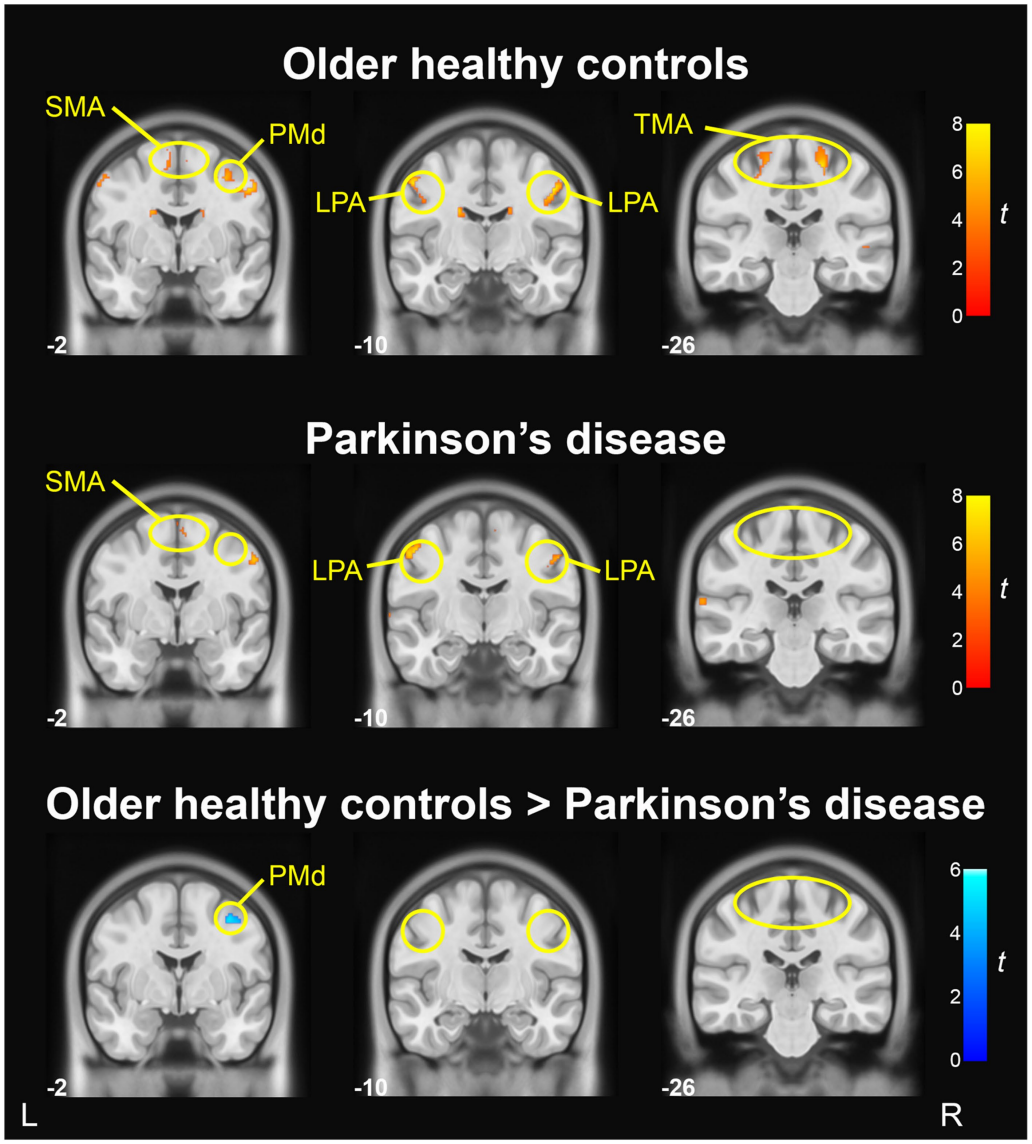
BOLD activation in primary motor and premotor cortices during sustained vowel production (Ah vs. Rest). BOLD activation maps show areas of significant activity within the older healthy control group (top), significant activity within the PD group (middle), and significant group differences in activity between the older healthy control group and Parkinson’s disease group (bottom); voxel-wise *p* < 0.001, uncorrected, cluster *p* < 0.05, uncorrected. Slice numbers reflect y coordinates in MNI space. Color bars correspond to t-statistics.

Table 5 reports the anatomical labels, size, coordinates, and t-statistics of all clusters meeting statistical significance. When comparing ‘Ah’ versus Rest conditions in the OHC group, BOLD activation in the motor cortices was found bilaterally in the LPA of the primary motor cortex (M1) and in the trunk motor area of M1, as well as right SMA and right dorsal premotor cortex (PMd; *p* < 0.001, uncorrected). Activation was also found bilaterally in the cerebellum (lobule VI), caudate, inferior frontal gyrus, and precuneus. In the right hemisphere, BOLD activity was found in superior temporal gyrus, cuneus, medial frontal gyrus, and insula. In the left hemisphere, additional BOLD activations were found in the inferior occipital gyrus and postcentral gyrus. Fewer areas of BOLD activation were found in the PD group when comparing ‘Ah’ versus Rest conditions. In the motor cortices, BOLD activity for the PD group was found in the bilateral LPA region of M1 and right SMA; however, there was no BOLD activity in the trunk motor area of M1 or in the right PMd (*p* < 0.001, uncorrected). Additional BOLD activations were found in the right cerebellum (lobule VI), right cuneus, left superior temporal gyrus, and left inferior occipital gyrus. Motor cortical activation maps for OHC and PD groups are shown in Figure 3 top and middle rows.

**Table 5:** BOLD fMRI activations during sustained vowel production (‘Ah’ vs. Rest).

Comparison	Brain region(s)	BA	Size (mm^3^)	MNI coordinates (peak)			*t*-value	Cluster *p*-value	
				x	y	z		*p*_uncorr_	*p*_FDR_
	BOLD activations								
OHC	R precentral gyrus (M1/LPA)	4/6	430	49	−7	41	8.5	<0.001	0.001
	R precentral gyrus (M1/TMA)	4/6	160	21	−25	59	7.63	0.002	0.029
	L cerebellum VI	–	370	−25	−63	−23	7.55	<0.001	0.001
	L caudate	–	224	−19	−13	27	7.5	0.001	0.01
	L precentral gyrus (M1/LPA)	4/6	364	−51	−9	47	7.22	<0.001	0.001
	R superior temporal gyrus	22/21	206	65	−35	5	6.56	0.001	0.013
	R precuneus	39/7	152	27	−61	35	6.24	0.003	0.03
	R cerebellum VI	17/18	410	15	−67	−17	6.13	<0.001	0.001
	R middle frontal gyrus (PMd)	6	202	35	−1	51	5.87	0.001	0.013
	L precentral gyrus (M1/TMA)	4	188	−17	−27	59	5.86	0.001	0.016
	L inferior occipital gyrus	17/18	124	−17	−91	−13	5.73	0.006	0.046
	R caudate	–	140	19	−13	25	5.7	0.004	0.038
	R superior temporal gyrus	22/21	110	55	−15	1	5.66	0.009	0.064
	R inferior frontal gyrus	44	132	49	13	17	5.63	0.005	0.042
	R cuneus	17/18	152	13	−95	−5	5.25	0.003	0.03
	R inferior frontal gyrus	46	126	37	33	17	5.19	0.006	0.046
	R superior frontal gyrus (SMA)	6	392	7	11	71	5.14	<0.001	0.001
	R inferior parietal lobule	40	240	49	−47	45	5.06	<0.001	0.008
	R medial frontal gyrus	8	134	9	17	51	4.59	0.005	0.042
	R caudate	–	64	19	31	1	5.31	0.038	0.219
	L inferior frontal gyrus	47/45	82	−35	31	5	5.25	0.021	0.14
	R insula	13	66	39	19	21	5.16	0.036	0.214
	L postcentral gyrus	3	72	−25	−35	57	5.06	0.029	0.184
	L precuneus	39	60	−27	−61	35	4.91	0.044	0.241
PD	R cerebellum VI	–	112	29	−65	−25	7.39	0.009	0.103
	L precentral gyrus (M1/LPA)	4/6	328	−51	−11	47	6.92	<0.001	0.003
	R precentral gyrus (M1/LPA)	6	312	55	−3	45	6.64	<0.001	0.003
	R superior frontal gyrus (SMA)	6	182	9	−5	63	5.11	0.001	0.028
	L superior temporal gyrus	42	122	−65	−25	13	5.04	0.007	0.099
	L inferior occipital gyrus	17/18	106	−17	−91	−11	4.66	0.01	0.103
	R cuneus	18	68	21	−97	7	4.82	0.033	0.286
OHC > PD	R middle frontal gyrus (PMd)	6	86	37	−1	51	5.28	0.019	0.319
PD > OHC	–	–	–	–	–	–	–		

Voxel-wise threshold *p* < 0.001, uncorrected; cluster threshold *p* < 0.05, uncorrected. BA, Brodmann area, M1, primary motor cortex, LPA, laryngeal/phonatory area, SMA, supplemental motor area; TMA, trunk motor area; PMd, dorsal premotor cortex.

### 3.5. Comparison of BOLD activity between OHC and PD groups

A vowel-wise comparison of OHC and PD groups showed that BOLD activity in right PMd was significantly lower in the PD group (*p* < 0.001, uncorrected; Figure 3 bottom row). However, when applying a more conservative cluster-level statistical threshold (*p* = 0.05, FDR-corrected), this finding did not reach statistical significance. There were no regions in which BOLD activity was higher in PD compared to OHC (Table 4).

### 3.6. Correlation of right PMd with voice measures

We examined behavioral correlations with right PMd by first extracting the mean beta values of right PMd for each participant. For the 12 PD participants who had CAPE-V ratings of dysphonia severity, we correlated right PMd activity with expert ratings of deviancy in the following domains: loudness, pitch, roughness, breathiness, and strain. Correlations were subjected to false-discovery rate (FDR) correction using the Bejanamini and Hochberg linear step-up procedure (Benjamini and Hochberg, 1995). Results of the correlation analyses indicated that right PMd beta values were negatively correlated with severity ratings in the loudness domain (*r* = −0.669, *p*_uncorr_ = 0.017, *p*_FDR_ = 0.043) and pitch domain (*r* = −0.731, *p*_uncorr_ = 0.007, p_FDR_ = 0.035) the CAPE-V. Right PMd beta values were not correlated with ratings of severity in the roughness (*r* = 0.215, *p*_uncorr_ = 0.503, *p*_FDR_ = 0.629), breathiness (*r* = −0.434, *p*_uncorr_ = 0.159, *p*_FDR_ = 0.265), or strain (*r* = 0.037, *p*_uncorr_ = 0.908, *p*_FDR_ = 0.908) domains of the CAPE-V.

**Figure 4:**
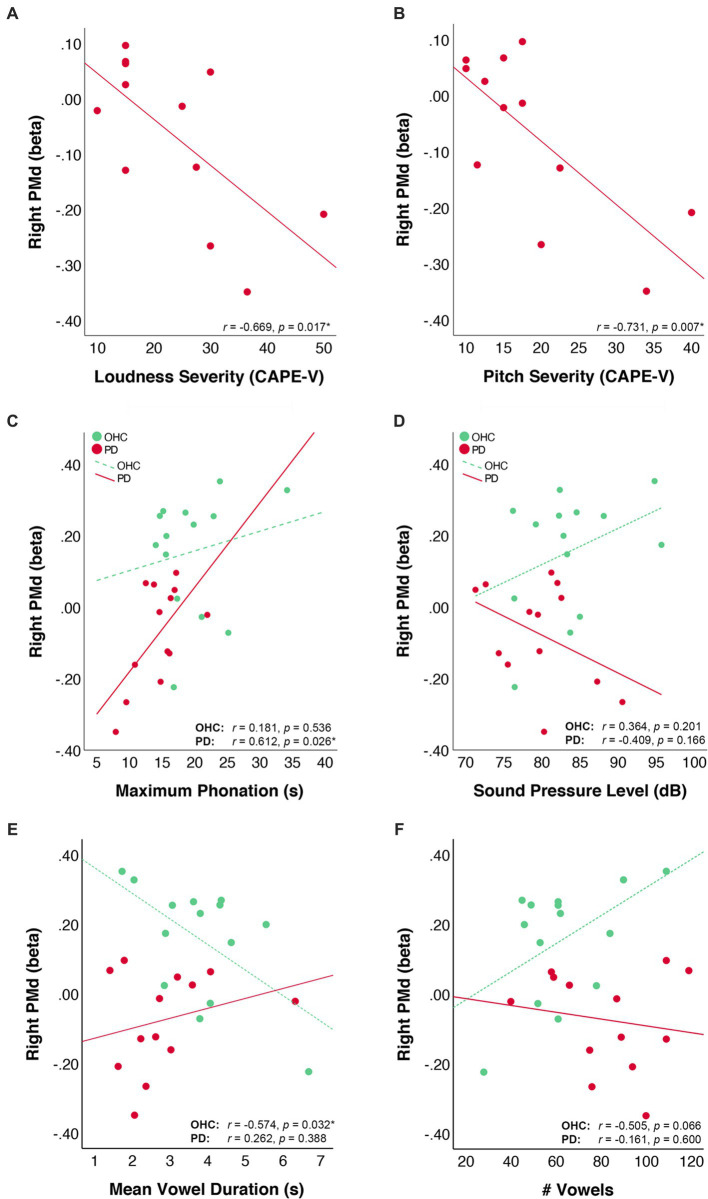
Correlation of right PMd activity values with voice measures. Scatter plots depict the correlation of right PMd beta values with **(A)** CAPE-V ratings of loudness severity, **(B)** CAPE-V ratings of pitch severity, **(C)** voice sound pressure level during sustained vowel production in a mock MRI scanner, and **(D)** maximum phonation time, **(E)** mean vowel duration in the scanner, and **(F)** total number of vowels produced in the scanner. Lines represent the linear fit of data points within each group. Data from the OHC group are plotted in green and data from the PD group are plotted in red.

Within both the PD and OHC groups, we also correlated right PMd beta values with the out-of-scanner acoustic measures of maximum phonation time, and voice SPL. There was a positive correlation between right PMd beta values and maximum phonation time in the PD group (*r* = 0.612, *p*_uncorr_ = 0.026, *p*_FDR_ = 0.052), but not the OHC group (*r* = 0.181, *p*_uncorr_ = 0.536, *p*_FDR_ = 0.536). There was no correlation between right PMd beta values and voice SPL in either the PD group (*r* = −0.409, *p*_uncorr_ = 0.166, *p*_FDR_ = 0.166) or OHC group (*r* = 0.364, *p*_uncorr_ = 0.201, *p*_FDR_ = 0.40). Scatter plots depicting the behavioral correlations for OHC and PD groups are depicted in Figure 4.

We further correlated right PMd values with measures of in-scanner task performance. For the OHC group, there was an inverse correlation between right PMd beta values and vowel duration (*r* = −0.574, *p*_uncorr_ = 0.032, *p*_FDR_ = 0.064) and a marginal positive correlation between right PMd beta values and number of vowels produced (*r* = 0.505, *p*_uncorr_ = 0.066, *p*_FDR_ = 0.066). For the PD group, PMd beta values did not correlate with either vowel duration (*r* = 0.262, *p*_uncorr_ = 0.388, *p*_FDR_ = 0.600) or number of vowels produced (*r* = −0.161, *p*_uncorr_ = 0.600, *p*_FDR_ = 0.600).

## 4. Discussion

The present study has four main findings. First, our acoustic measures showed that the PD group had reduced maximum phonation time compared to OHCs, and that the PD group tended to produce more vowels of shorter duration when completing the self-paced sustained vowel task. Second, individuals in the PD group recruited fewer brain regions during the sustained vowel production task compared to the OHC group. Most notably, activations in the bilateral trunk motor area and right PMd were present in the OHC group, but not in PD. Third, we found that BOLD activity in right PMd was significantly lower in the PD group compared to OHC. Fourth, we observed that lower BOLD activity in right PMd was associated with shorter maximum phonation time as well as higher CAPE-V ratings of loudness and pitch deviance in the PD group.

Acoustic measures of voice collected both inside and outside of the MRI scanner were indicative of reduced respiratory-phonatory support in the PD group. Outside of the scanner, we observed significantly lower maximum phonation times in the PD group compared to OHC. The maximum phonation time reported in the OHC group is consistent with published norms for older healthy adults (Maslan et al., 2011). In addition, the observation of lower maximum phonation time in PD supports previous studies (Yuceturk et al., 2002; Midi et al., 2008; Bauer et al., 2011) and may reflect reduced respiratory support for phonation and/or reduced glottal efficiency in PD. When participants performed the sustained vowel production task in a simulated fMRI environment, SPL values were higher than those typically observed during sustained vowel recording due to the effects of laying supine and listening to 90 dB acoustic noise (Manes et al., 2021). The magnitude of the SPL difference between the groups (−4.712 dB SPL) was comparable to the difference of −4.734 dB SPL reported in our previous paper; however, unlike our previous paper, the mixed effects model did not yield a significant effect of group on SPL. The lack of a significant group effect on SPL (*p* = 0.158) is in part due to the reduced power of fewer overall observations compared to our prior study. Furthermore, the previously reported group effects were smallest in the Mock Scanner + MRI noise condition, reflecting a group*condition interaction effect on voice SPL. While the SPL data from the Mock Scanner + MRI Noise condition were deemed to be the most ecologically similar to the fMRI task, on their own they were unable to capture group differences in voice intensity. When participants performed the sustained vowel production task in the scanner, we found that individuals in the PD group tended to produce more vowels of shorter duration compared to the OHC group. The shorter vowel duration in PD is likely the result of the decreased respiratory-phonatory support, in line with decreased maximum phonation time observed in this group. Individuals with PD may be self-selecting shorter phonation times to conserve respiratory/phonatory effort. Studies of speech breathing have found that individuals with PD have a faster resting tidal breathing rate and spend less time producing speech within a breath group (Solomon and Hixon, 1993). The observed duration and number of vowels produced by each group suggest that the performance of self-paced vowel productions is not identical between OHC and PD groups. However, given that there was no significant group difference in the total time spent vocalizing during the task, the tradeoff between vowel length and number of vowels produced likely balances out the overall level of BOLD activation within each block.

Overall, the PD group recruited fewer brain regions than the OHC group during the sustained vowel production task. Both OHC and PD groups exhibited activation of cortical and subcortical brain regions that have been reported in previous fMRI studies of phonation, including primary motor cortex (Ozdemir et al., 2006; Loucks et al., 2007; Brown et al., 2008, 2009; Peck et al., 2009; Bouchard et al., 2013; Belyk and Brown, 2017; Eichert et al., 2020; Belyk et al., 2021), SMA (Loucks et al., 2007; Brown et al., 2008, 2009; Parkinson et al., 2012; Grabski et al., 2013), cerebellum lobule VI (Brown et al., 2009; Belyk et al., 2021), and superior temporal gyrus (Ozdemir et al., 2006; Loucks et al., 2007; Brown et al., 2009; Peck et al., 2009; Parkinson et al., 2012; Belyk et al., 2021). However, the OHC group also exhibited activation in the caudate (Belyk et al., 2021), inferior frontal gyrus (Ozdemir et al., 2006; Loucks et al., 2007; Peck et al., 2009; Parkinson et al., 2012; Belyk et al., 2021), right insula (Peck et al., 2009; Parkinson et al., 2012), and right premotor cortex (Loucks et al., 2007; Wilson et al., 2011; Belyk et al., 2021).

With respect to motor cortical activity, SMA activity was present in both OHC and PD groups. However, there were differences in the recruitment of primary motor and premotor cortical areas during sustained vowel production. In the OHC group, we found activity bilaterally in two clusters along the precentral gyrus: (1) the dorsal LPA, which is purported to control the intrinsic laryngeal muscles (Brown et al., 2008; Simonyan, 2014; Correia et al., 2020), and (2) the trunk motor area, which is involved in volitional breathing (Ramsay et al., 1993; Takai et al., 2010) as well as phonation (Olthoff et al., 2008; Correia et al., 2020). By contrast, BOLD activation maps for the PD group showed activity in the bilateral dorsal LPA, but no activity in the trunk motor area. This absence of observed activity in the trunk motor area may reflect a reduced cortical drive for engaging the muscles of the chest wall during speech breathing (Hovestadt et al., 1989; Solomon and Hixon, 1993; Sabate et al., 1996). Reduced respiratory-phonatory support from the expiratory muscles of the trunk would be consistent with our finding of reduced maximum phonation time in PD. However, this would need to be examined in greater detail using more sophisticated measures of respiratory physiology.

Activity in the right PMd was found in the OHC group, but not in the PD group when performing within-group ‘Ah’ versus Rest contrasts. Further, the group comparison of BOLD activity in OHC vs. PD contrast demonstrated that the PD group had significantly lower activity in right PMd compared to OHC. Hypoactivity of right PMd has not been previously reported during speech production in PD. However, a study by Narayana et al. (2020) reported that PD participants with hypokinetic dysarthria had less activity in left PMd compared to typical speakers when performing a connected speech task during both PET and fMRI. The authors also noted that while both the PD group and control group recruited bilateral PMd during the fMRI connected speech task, the right hemisphere cluster was much smaller in the PD group. Moreover, while the control group recruited both left and right PMd when performing the speech task during PET, the PD group recruited only left PMd. Although hypoactivation in right PMd has not been previously reported in PD during speaking tasks, our finding is in line with prior work examining the neural correlates of voice treatment in PD. Narayana et al. (2010) found that PD participants had activation in right PMd only after completing the LSVT program. Further, the activity in right PMd was positively correlated with increased voice intensity (SPL) following successful voice treatment (Narayana et al., 2010). As the participants in our PD group had not completed the LSVT program within the past 2 years, the absence of right PMd activity in our study is consistent with the findings observed in the pre-treatment group. It also suggests that reduced activity in PMd is a potential biomarker for therapeutic intervention.

While the precise role of right PMd in speech production is unclear, there is growing evidence that PMd plays a key role in phonatory control. Indeed, neuroimaging studies have reported activation of the right PMd during singing (Saito et al., 2006; Wilson et al., 2011; Belyk et al., 2021), humming (Hickok et al., 2003), and vowel production (Loucks et al., 2007), as well as during volitional exhalation (Loucks et al., 2007). Furthermore, our right PMd cluster appears to overlap with the ‘dorsal precentral speech area’ (dPCSA), which has been proposed by Hickok et al. (2023) to serve as a region for coordinating pitch-related vocalizations, such as song and prosody. Hickok et al. (2023) propose that the dPCSA (PMd) is part of a hierarchical system responsible for planning and coordinating pitch-related vocalizations *via* projections to the dorsal LPA. Indeed, evidence from younger healthy adults suggests that right PMd is one of several areas which exhibit functional connectivity with the LPA during voice production (Simonyan et al., 2009). In the present study, we found no differences in LPA activity between or OHC and PD groups, suggesting that phonatory symptoms in PD may be driven more by changes in the preparatory input to LPA rather than changes in the LPA itself.

In addition to its proposed role in phonatory control, it is also worth considering the potential role of right PMd in self-perception during phonation. A number of functional imaging studies have shown that PMd is active during both speech production and perception tasks (Skipper et al., 2005; Meister et al., 2007; Callan et al., 2010; Tremblay and Small, 2011; Glanz Iljina et al., 2018) - although we note that some studies refer to this area as “superior PMv” rather than PMd. Moreover, cortical stimulation studies have shown that premotor cortex is critical for the self-perception of motion or ‘motor awareness’ (Desmurget and Sirigu, 2009; Bolognini et al., 2016; Fornia et al., 2020; Marotta et al., 2021). For example, disruption of right PMd activity *via* cathodal transcranial direct current stimulation (tDCS) impaired the participants’ ability to reliably estimate their own motor performance (Bolognini et al., 2016). Given that people with PD have poorer self-awareness of their own voice intensity (Ho et al., 1999, 2000) and abnormal monitoring of their own sensory feedback during phonation (Liu et al., 2012; Mollaei et al., 2016), it is possible that the hypoactivation we observed in right PMd reflects an impaired motor awareness for phonation in PD.

Our correlation analyses showed that PD participants with lower levels of right PMd activity had more deviant perceptual ratings of voice loudness and pitch on the CAPE-V. The inverse correlation between right PMd and loudness deviance is in line with the findings of Narayana et al. (2010), showing that right PMd activity was positively correlated with increased voice intensity following successful voice treatment. Engaging right PMd during overt speech and vowel production tasks may reflect more purposeful or effortful phonation to compensate for disease-related impairments in automated scaling of voice intensity. The correlation of PMd with pitch deviance is in line with the hypothesis that PMd is involved in the coordination of vocal pitch (Hickok et al., 2023). The fact that our right PMd cluster was correlated with both loudness and pitch deviation measures could point to a slightly broader impairment of coordinating the laryngeal musculature for phonatory control in PD. For example, engaging right PMd during phonation may help to modulate tension in the vocal folds for both loudness and pitch modulation. Since vocal fold atrophy is a feature of both typical aging (Ramig et al., 2001a) and PD (Ma et al., 2020), the need for premotor activity may reflect an increased effort required for generating or maintaining vocal fold tension during phonation. In the case of PD, dysregulation of the cortico-basal ganglia motor loop may impair the ability to effectively engage PMd for increasing vocal fold tension, resulting in hypophonia as well as changes in pitch modulation.

In addition to our perceptual measures, we found that activity in right PMd was positively correlated with maximum phonation time in the PD group. If our right PMd cluster is involved in the modulation of laryngeal muscle activity, its association with longer maximum phonation times may be a function of increased glottal efficiency. Impaired glottal closure is associated with difficulty sustaining phonation as well as reduced voice intensity in PD (Hanson et al., 1984). It is possible that engaging right PMd can help to achieve more glottic closure for increasing phonation time as well as increasing loudness. An alternative interpretation is that right PMd is involved with generating and maintaining a respiratory drive for sustained phonation in PD. The evidence for this is somewhat tempered by the lack of a significant correlation between in-scanner vowel duration and PMd activity in the PD group. However, we also note that participants were instructed to produce vowel durations of 2–3 s in the scanner and that these shorter vowel production times do not necessarily reflect the full range of respiratory-phonatory capacity that is captured by maximum phonation times. Given that the correlation between PMd and maximum phonation time was marginal after applying FDR correction, the effect should be interpreted with caution until replicated in a larger sample.

In contrast to the PD participants, right PMd activity in the OHC group was not correlated with maximum phonation time. Rather, right PMd activity in OHCs was inversely correlated with vowel duration and marginally correlated with the number of vowels produced during the sustained phonation task. We note that neither of these correlations were statistically significant upon FDR correction; however, the observation suggests a trend for right PMd activity to be higher for OHC participants who self-selected to produce shorter, more frequent vowels. It may be the case that OHCs utilized right PMd for initiating, rather than sustaining, the vowel productions. If so, this would be in contrast to the PD group, who showed no relationship between right PMd activity and either vowel duration or number of vowels produced. Given the self-paced nature of the sustained vowel production task, we expected that movement initiation would be driven primarily by SMA rather than the lateral premotor cortex (Lu et al., 2012). Still, it is possible that PMd played a role in vowel initiation or received downstream modulation by SMA for initiating vowel production.

Interestingly, activity in our right PMd cluster was not correlated with SPL measures taken from our mock scanner recordings. While we sought to make our mock scanner environment as comparable to the fMRI environment as possible (Manes et al., 2021), and counterbalanced the order of testing, we cannot rule out the possibility that the participants behaved differently in our mock scanner and fMRI sessions. The fact that our right PMd cluster was inversely correlated with perceptual ratings of loudness deviance suggests that activity in right PMd is indeed related to perceived changes in loudness in PD. However, it will be important to replicate this finding in future studies using additional acoustic and perceptual measures.

While the present study points to a potential role of hypoactive right PMd in PD hypophonia, the findings should be interpreted cautiously, as the group differences in right PMd were not statistically significant when correcting for multiple comparisons. Further research will be needed to confirm whether hypoactivation of right PMd is associated with respiratory-phonatory symptoms in PD hypophonia. To fully understand the contributions of right PMd to phonatory control in PD, it will be important to investigate whether PMd activity scales with increasing respiratory-phonatory effort. Collecting more direct assessments of respiratory physiology during a phonatory fMRI task would help to assess whether there is a link between hypoactivity in the PMd and changes in respiratory drive or timing for phonation. Correlating PMd activity with measures of prosody in PD will also be a useful extension of the present research in light of the proposed role of PMd in pitch-related vocal control. In addition, it will be important to ascertain what role right PMd plays within the broader networks controlling vocalization. For example, future studies will be needed to investigate whether right PMd serves as a preparatory area for modulating the dorsal LPA, consistent with the model proposed by Hickok et al. (2023), or whether the influence of PMd on phonation in PD may be independent of the dorsal LPA. Investigating whether PMd is modulated by input from SMA will also provide insight into the possible role of PMd for coordinating voice initiation.

Collectively, our findings suggest that PD hypophonia is associated with functional changes in the right premotor cortex. Hypoactivation of right PMd may be related to decreased respiratory-phonatory support for pitch and loudness regulation in PD hypophonia.

## Data availability statement

The datasets presented in this article are not readily available because the datasets generated for this study are only available to the study investigators, per the privacy and data sharing policies listed in the consent form at the time of the study. Requests to access the datasets should be directed to JM, jmanes@bu.edu.

## Ethics statement

The studies involving humans were approved by Northwestern University Institutional Review Board. The studies were conducted in accordance with the local legislation and institutional requirements. The participants provided their written informed consent to participate in this study.

## Author contributions

JM contributed to the conception and design of this study, programmed the experimental and data analysis scripts, collected all primary data, including voice samples and neuroimaging data, performed the statistical analysis of the fMRI data, and wrote the first draft of the manuscript. AK and TP contributed to the design of the fMRI scanning protocols. JM and EH contributed to participant recruitment, screening, and administration of the MDS-UPDRS and contributed to the analysis of acoustic voice measures. KT, AR, and JM contributed to the selection and interpretation of voice measures. AR contributed to the collection and analysis of perceptual voice ratings. JM, EH, and AK contributed to the pre-processing of fMRI data. DC contributed to the editing, style, and structure of the manuscript. All authors contributed to the article and approved the submitted version.

## Footnotes

^1^https://audacityteam.org/

^2^https://cibsr.stanford.edu/tools/human-brain-project/artrepair-software.html
